# Interactive effects of arbuscular mycorrhizal fungi and organic amendments on maize growth under salinity stress

**DOI:** 10.3389/fpls.2026.1638742

**Published:** 2026-03-06

**Authors:** Khaled D. Alotaibi

**Affiliations:** Department of Soil Science, King Saud University, Riyadh, Saudi Arabia

**Keywords:** antioxidant activity, arbuscular mycorrhizal fungi, bio-inoculants, organic amendments, plant growth, salinity, Zea mays

## Abstract

Soil salinity poses a serious threat to sustainable crop production, particularly in arid and semi-arid regions. To mitigate its adverse effects, effective approaches, including the use of organic amendments and bio-inoculants have been sought as potential solutions. This study investigated the individual and combined effects of arbuscular mycorrhizal fungi (AMF), biochar (BC), and compost (CP) on the growth and morphological and physiological traits of maize (*Zea mays* L.) grown in saline and non-saline soils. Treatments included AMF, BC, CP, AMF+BC and AMF+CP in addition to unamended controls. Soil salinity significantly reduced maize growth, photosynthetic pigment content, and nutrient uptake, while increasing sodium accumulation and oxidative stress markers. Individual application of AMF, BC, and CP alleviated several salinity-induced constraints; however, their combined application showed higher and more consistent improvements. In particular, AMF combined with BC markedly enhanced root colonization, shoot and root biomass, chlorophyll content, and uptake of nitrogen, phosphorus, and potassium, while reducing sodium accumulation and improving the K:Na ratio under saline conditions. These improvements were associated with increased proline accumulation and activation of antioxidant enzymes (superoxide dismutase, catalase, peroxidase, and ascorbate peroxidase), leading to reduced levels of malondialdehyde and hydrogen peroxide. Principal component analysis further revealed strong positive associations between combined AMF–organic amendment treatments and growth, nutrient balance, and stress-defense traits under salinity. Overall, the results demonstrate that integrating AMF with organic amendments, particularly biochar, provides an effective strategy for enhancing maize tolerance to salinity through coordinated improvements in nutrient acquisition, ion homeostasis, and oxidative stress regulation. This study highlights the potential of AMF and organic amendment combinations as sustainable and cost-effective tools for managing salinity stress in maize production systems, especially in salt-affected arid environments. However, more research is necessary to validate these findings across different saline soils and crops in field conditions.

## 1 Introduction

Soil salinity has become a major focus of studies in the present climate change scenario, due to its significant impacts on plant growth and productivity, causing serious yield losses (Munns and Tester, 2008; Omar et al., 2023; Demo et al., 2025). It is estimated that by 2050, soil salinity is expected to become more severe and affect 50% of all arable land in large areas of the planet, highlighting its global threat to agricultural production (Shabala, 2013) and agroecosystems (Dar et al., 2022). Salts within soil induce morphological, physiological, and biochemical changes, which are interconnected and intervene directly or indirectly to the multiple plant functions (Assaeed et al., 2023; Malik et al., 2022b; Rahman et al., 2024). The morphological changes include a reduction in the production and growth of plants through several physiological responses, including alteration of osmotic setup and imbalanced ionic concentrations (Farooq et al., 2015). Soil salinity leads to excessive absorption of toxic Na^+^ ions, triggering excessive generation of hydrogen peroxide (H_2_O_2_) and hydroxyl radicle (O^-^) (Mangal et al., 2023). Accumulating ROS causes potential damage at the subcellular level that eventually affects plant growth (Desoky et al., 2020). Additionally, the abundant Na^+^ ions in solution compete with essential K^+^ ions, resulting in a decreased potassium to sodium ratio (K:Na) causing ionic toxicity, which damages the cellular structure and leads to deficiencies in water status, mineral nutrition, photosynthetic efficiency, and oxidative processes (Islam et al., 2023; Zhang et al., 2018).

The negative effects of salinity on plant growth and production have led to increased attention on mitigation strategies to counter its long-term effect on plant survival and yield (El-Akhdar et al., 2025; Zaib et al., 2023). Although various innovative methods, such as genetic engineering and plant breeding, have been developed to lessen the harmful effects of soil salinity on agricultural production and to breed salt-tolerant plant varieties, their progress has been slow and these techniques are generally expensive (Desoky et al., 2020). Furthermore, to avoid the impact of salt-induced oxidative damage and other salt-related deleterious effects, plants have developed protective mechanisms. These adaptive defenses mainly work at the cellular level and include the upregulation of antioxidant enzymes and osmolyte accumulation, such as proline (Rajput et al., 2021; Sharma et al., 2012). However, not all plants (e.g., glycophytes) possess these endogenous salinity defense mechanism(s). Maize (*Z. mays* L.) is one such glycophyte and holds a very essential role in global food production (Zhang et al., 2018). It is a C4 plant belonging to the *Poaceae* family. Soil salinity is one of the most common stresses in maize cultivationas it’s hypersensitive to salinity stress (Farooq et al., 2015). Therefore, maize cultivation in saline soils requires the exogenous application of growth-promoting agents to enhance its salt adaptive mechanisms. The role of arbuscular mycorrhizal fungi (AMF), organic amendments, such as biochar (BC) and compost (CP), for use in mitigating salinity stress is well-established.

The obligate symbiotic association developed between the endosymbiotic rhizospheric AMF and terrestrial plants is widespread, representing approximately 80% of land plants (Smith and Read, 2010). Such AMF symbiosis has been reported to alleviate the salt stress for a wide range of crop and forage plants (Malik et al., 2024; Peng et al., 2024). For example, this AMF interaction enable plants to expand their root absorption range, improving their capacity to acquire water and essential mineral nutrients, especially in saline soils (Parihar et al., 2020). Moreover, the symbiotic relationship with these biotrophic fungi also upregulate various plant physiological processes, enhancing photosynthetic efficiency by increasing the levels of photosynthetic pigments (Hamzehzadeh et al., 2024). These AMF associations also boost antioxidant enzyme activity (Han et al., 2024; Malik et al., 2022a; Wang et al., 2022).

Utilization of BC and organic CP have also been reported to mitigate salinity stress (Mbarki et al., 2020; Patel and Panwar, 2023). The application of these biofertilizers alleviate the salinity stress by improving rhizosphere conditions of soil, enhancing soil water holding capacity, and improving soil fertility (Cui et al., 2021; Mbarki et al., 2018; Naeem et al., 2017). Additionally, BC can facilitate ion-exchange reactions and decrease Na^+^ bioavailability; thereby, reducing its toxicity and ROS abundance (Blackwell et al., 2010; Nguyen et al., 2022; Yu et al., 2023). CP, on the other hand, improves soil nutrient content, enhances photosynthesis and boosts antioxidant defense system (Abo-Elyousr et al., 2022; Lakhdar et al., 2008; Trivedi et al., 2017). Previous studies suggest that crops treated with AMF+BC and AMF+CP amendments develop advanced mechanisms to support plant growth under salt stress (Gunes et al., 2023; Mbarki et al., 2018). This is achieved by enhancing the AMF benefits via BC or CP feedback effects that improve photosynthetic activities, antioxidant enzyme activity, soil N, P, and K uptake, along with increasing proline content (Ait-El-Mokhtar et al., 2020; Gunes et al., 2023; Kazemi et al., 2019; Ouhaddou et al., 2022).

Although AMF, BC, and CP have been studied individually, their combined effects on maize under saline conditions remain poorly understood. To fill this gap, this study investigates the combined interaction of AMF, BC and CP. This study hypothesizes that AMF added to BC and CP improves plant salinity adaptation more than individual treatments. The objectives were to (1) evaluate individual and combined treatments on maize growth and stress indicators under salinity. (2) evaluate chlorophyll content, proline accumulation and antioxidant enzyme activity and (3) determine how each amendment treatment improved nutrient uptake. By addressing these objectives, this study provides innovative insights into eco-friendly, cost-effective strategies for sustainable crop production in salt-affected soils, with a particular emphasis on arid and semi-arid regions, by understanding the integrated effect of AMF, BC and CP.

## 2 Materials and methods

### 2.1 Preparation of AMF inoculum, biochar and compost

The AMF inoculum used in this experiment was sourced from the rhizosphere of *Olea europaea* L. cultivar ‘Picual’ growing in the Aljouf region of Saudi Arabia (coordinates). The orchard soil is naturally saline with an EC of 5.98 ± 0.35 *dS m^-1^*. The spores were extracted from the collected samples by following the method of Boyno et al. (2023). The isolated spores were classified based on morphological characteristic descriptions i.e., color, wall structure, shape, and surface ornamentation (Schenck, 1990) and compared with the classifications manuals described by the International Culture Collection of Vesicular-Arbuscular Mycorrhizal Fungi (INVAM: http://invam.ku.edu/species-descriptions). Identification descriptions of Schüßler et al. (2001) and Redecker et al. (2013) were also followed which resulted in the predominant occurrence of species: *Entrophospora etunicata* (W.N. Becker & Gerd.) Błaszk., Niezgoda, B.T. Goto & Magurno, 2022 (Błaszkowski et al., 2022)*, Funneliformis geosporum* (T.H. Nicolson & Gerd.) Schüßler and Walker, 2010, and *Funneliformis mosseae* (T.H. Nicolson & Gerd.) Schüßler and Walker, 2010 (Schüßler and Walker, 2010). The spores were subsequently multiplied in a trap culture methodology. The trap culture contained field AMF soil mixed with sterilized sand (1:2 w/w) with maize as host plant. The setup was maintained in a greenhouse of College of food and Agriculture Science with controlled environmental conditions with 14 h photoperiod and a temperature of 20/30 °C N/D. The trap culture was harvested after five months and the soil substrate was used as inoculum and contained a consortium of AMF spores (*E. etunicata* = 16 spores g^-1^ dry soil; *F. geosporum* = 11 spores g^-1^ dry soil; and *F. mosseae* = 19 spores g^-1^ dry soil), infected roots (73%) and hyphae.

The BC was produced at a pyrolysis temperature of 500°C using soft offal meal, a byproduct of poultry processing industry, as a feedstock. The soft offal meal was placed in a tightly closed chamber, transferred to a muffle furnace, and pyrolyzed for 3 h. The BC was ground to pass through a 1-mm sieve. The CP was sourced from commercially available composted cattle manure. Prior to their use, the BC and CP were analyzed (**Table 1**).

**Table 1:** Basic characteristics of the biochar and compost used in the experiment.

Amendment	pH	EC	Total C	Total N	Total P
		ds m^-1^	%		
Biochar	9.61	2.60	44.81	9.89	6.95
Compost	8.74	10.73	26.62	0.95	0.60

### 2.2 Soil and seed preparation

The soils used for the experiment were collected from two peach (*Prunus persica* L.) orchards in AlJouf province, located in the northeastern region of Saudi Arabia. The two soils differed in their salinity levels and were classified as saline or non-saline, based on their EC values (**Table 2**). The collected soil samples were air-dried, ground, and passed through a 2-mm mesh sieve and stored at room temperature until use. Furthermore, before using the soil in the experiment, the presence and viability of AMF propagules in both soil types were verified. The results confirmed the presence of 14 (dead) spores g^-1^ dry saline soil and 9 (dead) spores g^-1^ dry non-saline soil and the absence of any viable spores and colonization structures in the roots. Therefore, the presence of AMF in the study soil was neglected in the experimental setup. Before the initiation of the experiment, both soils were analyzed for basic physicochemical properties (**Table 2**) of each soil type were analyzed following standard procedures of Bowles (1992) for soil texture, Rowell (2014) for EC and pH and Bremner and Mulvaney (1982) for total nitrogen (N) content. The remaining portion of each soil type was mixed with AMF (2% w/w or 10.6 g), BC (1% w/w or 5.3 g), and CP (1% w/w or 5.3 g) and their combinations (2:1 w/w), then placed into pots with a soil capacity of 530 g and dimensions 6 (top) X 15 (length) X 5 (bottom) cm. The control untreated pots received the same amount of autoclaved AMF inoculum.

**Table 2:** Selected physicochemical properties of soil used in the experiment.

Soil type	pH (1:2)	E.C. (dS m^-1^)	OM (%)	Total N (%)	Clay (%)	Silt (%)	Sand (%)	Texture
Non-Saline	8.11	0.24	1.13	0.047	9.2	7.1	83.7	loamy fine sand
Saline	7.71	8.35	1.34	0.068	8.6	7.7	83.7	loamy fine sand

The maize (*Z. mays* L., hybrid-310) seeds were sourced from the Plant Production Department, College of Food and Agriculture Sciences, King Saud University. Maize generally exhibits substantial genetic variation in its tolerance to salinity stress as some salt-tolerant maize hybrids have been successfully developed, but hybrid-310 remains sensitive to salinity (Seleiman et al., 2023). The seeds were surface-sterilized by soaking in 95% ethanol for 30 sec, and 5% sodium hypochlorite for 10 min, then thoroughly rinsed with distilled water and soaked for 24 h in the dark. The seeds were subsequently germinated in petri dishes lined with double sterilized Whatman No. 1 filter paper. The uniformly germinated seeds were transplanted in pots (three seedlings per pot) containing AMF, BC, CP, AMF+BC, or AMF+CP amended saline and non-saline soil, with untreated control. The untreated control for both saline and non-saline soil was added to establish a baseline for comparison with the effect of bio-inoculant amendments.

### 2.3 Growth conditions and experimental design

The experiment was conducted in the greenhouse of the College of Food and Agriculture Sciences, King Saud University, Riyadh. The climate conditions of the greenhouse were maintained as follows: The day/night cycle 14/10 h with average day/night temperatures of 25 ± 2/20 ± 2°C and relative humidity of ~60%. The experiment was a two-factor factorial setup carried out in a completely randomized design arrangement of pots. The first factor was soil type: (saline and non-saline), and the second factor was amendment: none (i.e., control), AMF, BC, CP, AMF+BC, and AMF+CP. A total of 48 pots (i.e., 2 soils types x 6 amendments x 4 replications) were used, with maize seedlings transplanted in each pot and grown for five months. Soil moisture was maintained at 80% of field capacity for the entire period of study. A full-strength Hoagland’s nutrient solution (without P) was applied every 15 days throughout the experiment.

### 2.4 Symbiotic development and plant growth parameters

After harvesting the plants, their fine roots were carefully separated and stained with trypan blue (Phillips and Hayman, 1970). Thirty 1-cm fine root segments were then observed under a microscope to determine the percentage rate of AMF colonization (McGonigle et al., 1990). At harvest, the shoot and root systems were separated and the shoot length (SL), root length (RL), shoot dry weight (SDW), and root dry weight (RDW) were determined. The number of leaves/plant (L/P), and basal stem diameter (BSD; measured by using a Vernier Caliper) were recorded before the plants were harvested.

### 2.5 Photosynthetic pigments

The concentration of photosynthetic pigments (Chlorophyll*_a_*, chlorophyll*_b_*, total chlorophyll, and carotenoids) were quantified by following the method described by Arnon (1949). Briefly, the process consisted of initially grinding 100 mg of fresh leaves in 10 mL of 80% acetone. The resulting homogenate was centrifuged at 5000 g for 5 min, using a Benchtop Centrifuge-5810R (Eppendorf, Hamburg, Germany). The samples were then incubated in the dark for three hours and subsequently analyzed for absorbance at wavelengths 480, 510 nm for carotenoid calculation and 645, and 663 nm for chlorophyll*a*, *b* and total, using a UV-VIS spectrophotometer (SHIMADZU, Kyoto, Japan, UV1800). The concentrations (ug g^-1^fresh weight) of Chlorophyll*_a_*, chlorophyll*_b_*, total chlorophyll, and carotenoids were then calculated using the equations below:


$$Chlorophyll\;a=(\;\frac{12.7(A663)-2.69(A645)\;X\;V}{1000})\;W$$

$$Chlorophyll\;b=(\;\frac{22.9(A645)-4.68(A663)\;X\;V}{1000})\;W$$

$$Total\;Chlorophyll\;=(\;\frac{20.2(A645)+8.02(A6663)\;X\;V}{1000})\;W$$

$$Carotenoid=(\;\frac{7.6(A480)-1.49(A510)\;X\;V}{1000})\;W$$

### 2.6 Plant nutrient content

A subsample of the oven-dried aboveground biomass was finely ground and acid-digested according to the method detailed by Thomas et al. (1967), after which nitrogen (N), phosphorus (P), potassium (K) and sodium (Na) were determined. The Kjeldahl method was used to determine N following the method described by Jones (1991), whereas the P was measured colorimetrically following the procedure described by Murphy and Riley (1962). The K and Na were measured using a flame photometer (Rhoades, 1983). Plant P and N uptake were calculated by multiplying the tissue P and N content by their corresponding dry weight.

### 2.7 Biochemical traits and antioxidant enzyme activity

The proline (µg g^-1^ FW) was estimated by following the established procedure of (Carillo et al., 2008).

The malondialdehyde (MDA) content was estimated by following the methods of Esterbauer and Cheeseman (1990), while the hydrogen peroxide (H_2_O_2_) content in the maize tissue was measured using the method of MacNevin and Urone (1953). For measuring antioxidant enzyme activity, 100 mg of frozen plant tissue were ground in 4 mL of 1M phosphate buffer (pH 7) containing 5% polyvinylpolypyrrolidone. The homogenate was then centrifuged at 18000 g for 15 minutes at 4°C, and the supernatant was collected to assess antioxidant enzyme activity (García et al., 2004). The superoxide dismutase (SOD) activity expressed as (U mg^-1^protein), was assayed by the method of Beyer Jr and Fridovich (1987). The catalase (CAT) activity (U mg^-1^protein) was measured by following the method of Aebi (1984). The ascorbate peroxidase (APX) activity (U mg^-1^protein), was assayed as a decrease in absorbance at 290 nm for 1 min according to Amako et al. (1994). The peroxidase (POD) activity (U mg^-1^protein), was determined by monitoring the change in absorbance at 470 nm using the guaiacol test (Polle et al., 1994).

### 2.8 Statistical analysis

A two-way analysis of variance (ANOVA) using factorial design was conducted to ascertain the statistically significant differences induced by the soil type, bio-inoculants and their interactions. using the Mixed procedure of SAS (version 9.2; SAS Institute, Inc. Cary, NC; Littell et al., 2006). The data was subjected to the arcsin transformation when necessary to guarantee uniformity of variance. When a significant difference was found, Tukey’s Honestly Significant Difference (HSD) test at (p ≤ 0.05) was carried out to establish significant differences among mean treatments. A principal component analysis (PCA) was conducted using JMP version 2016 Pro software (SAS Institute, Inc., Cary, NC, USA), to investigate the interaction among the parameters and different soil amendments. The cosine of the angle between variable vectors indicates the correlation strength among variables, with variable groupings arbitrarily based using an angle of 40° (i.e., r = 0.77; Hangs et al., 2014). Variable vectors pointing in the same direction represent direct relationships, opposing vectors indicate indirect relationships, and unrelated variables have vectors perpendicular to each other.

## 3 Results

### 3.1 Corn root AMF colonization

**Figure 1:**
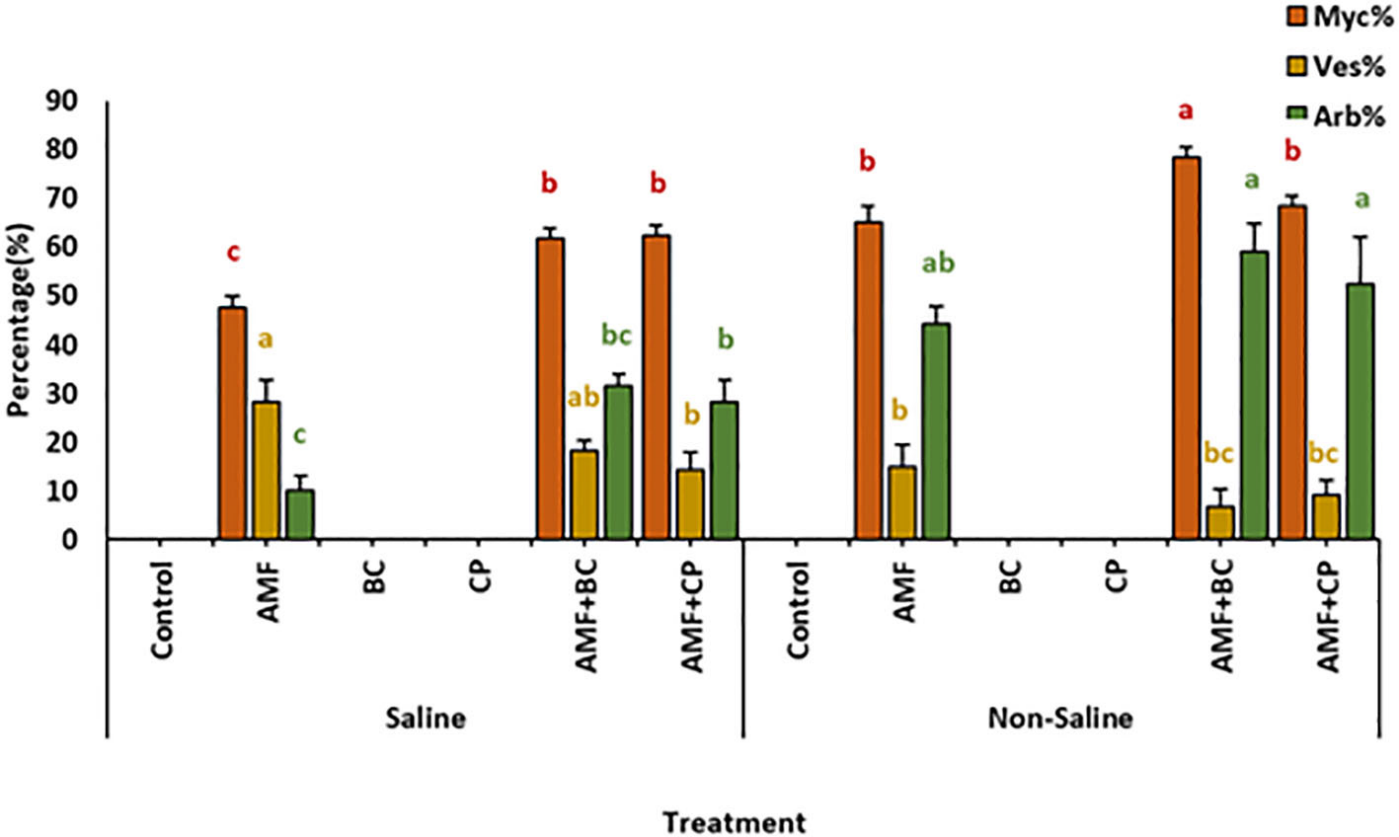
Root colonization of maize by arbuscular mycorrhizal fungi (AMF), showing mycelium (Myc), vesicles (Ves), and arbuscules (Arb), in saline and non-saline soils amended with biochar (BC) and compost (CP). Bars represent mean values ± standard error (n = 4). Different letters indicate significant differences among treatments according to Tukey’s HSD test (p ≤ 0.05).

Detailed analyses of the AMF root colonization showed that the formation of mycelium, vesicles and arbuscules was clearly higher in the corn roots grown in non-saline soils than in the saline soils (**Figure 1**). This indicates that soil salination had a significant negative effect on root colonization by AMF. However, amending saline and non-saline soil with BC and CP induced an increase in root colonization. In saline soils, the mycellium formation reached to ~62.5 ± 2.1 and ~61.6 ± 2.1% in plants treated with AMF+CP and AMF+BC, respectively, while in non-saline soil, mycelial growth rose to ~78 ± 2.1% in AMF+BC treated plants. Similarly, percentage formation of vesicles and arbuscules in corn roots showed a notable increase when treated with AMF+BC and AMF+CP than the AMF inoculated roots in both saline as well in non-saline soils. Microphotographs showing the colonization in the roots of maize in saline and non-saline soils is provided in a **Supplementary Figure (S1)**.

### 3.2 Corn plant morphology

**Figure 2:**
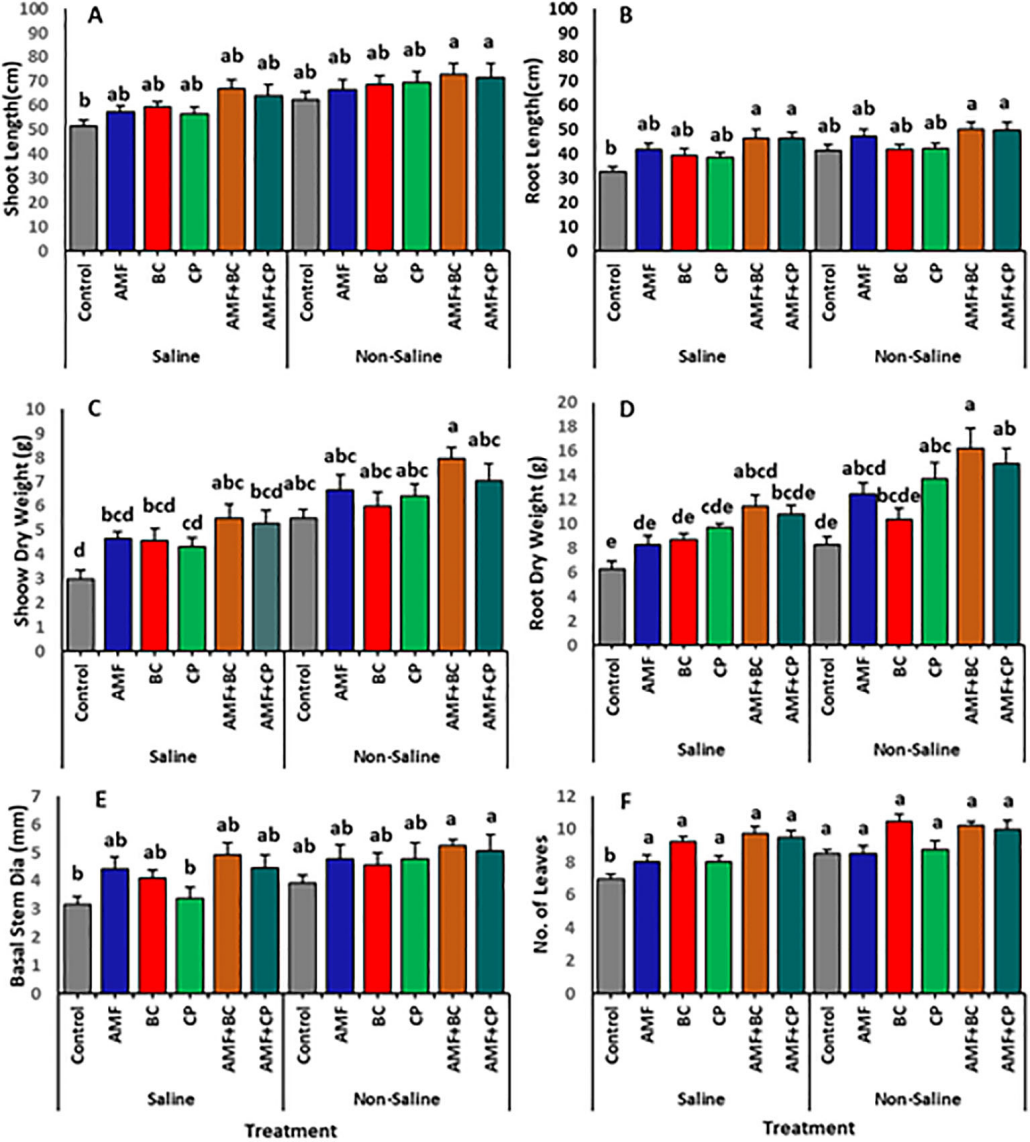
Effects of arbuscular mycorrhizal fungi (AMF), biochar (BC), compost (CP), and their combinations (AMF+BC and AMF+CP) on morphological parameters of maize grown in saline and non-saline soils: **(A)** shoot length (SL), **(B)** root length (RL), **(C)** shoot dry weight (SDW), **(D)** root dry weight (RDW), **(E)** basal stem diameter (BSD), and **(F)** number of leaves per plant (L/P). Bars represent mean values ± standard error (n = 4). Different letters indicate significant differences among treatments according to Tukey’s HSD test (p ≤ 0.05).

Soil salinity adversely impacted the corn morphology, such as SL, RL, SDW, and RDW, with no influence on BSD and L/P (**Table 3**; **Figure 2**). The combined application of AMF+BC and AMF+CP often mitigated the negative effects of salinity on the growth parameters. Plants treated with the AMF+BC amendment exhibited the highest SL, showing a ~16% increase relative to the untreated control plants. Similarly, the SL of AMF+BC treatment in saline soil showed an increase of 7% and 30% compared to control plants under non-saline and saline conditions, respectively (**Figure 2A**). The highest RL was depicted by the plants inoculated with AMF+BC (Saline plants = 46.5 ± 3.7 and non-saline plants = 50.3 ± 2.9 cm) and AMF+CP (saline plants = 46.7 ± 2.3 and non-saline plants = 49.8 ± 3.2 cm) than the control plants under both saline and non-saline environments (**Figure 2B**).

**Table 3:** Analysis of variance (factorial design) of morphological parameters of maize plants in saline and non-saline soil with different treatments of AMF, BC, CP, and their combination.

Morphological studied parameters	Source of variation (ANOVA)	P value
Shoot length (cm)	Soil Type (S)	0.0002***
	Soil Amendment Treatments (T)	0.0417*
	S x T	0.955ns
Root Length (cm)	Soil Type (S)	0.004**
	Soil Amendment Treatments (T)	0.0003***
	S x T	0.832ns
Shoot Dry Weight (g)	Soil Type (S)	<0.0001***
	Soil Amendment Treatments (T)	0.0006***
	S x T	0.885ns
Root Dry Weight (g)	Soil Type (S)	<0.0001***
	Soil Amendment Treatments (T)	<0.0001***
	S x T	0.538ns
Stem Dia (mm)	Soil Type (S)	0.011*
	Soil Amendment Treatments (T)	0.015*
	S x T	0.788ns
Leaves (No.)	Soil Type (S)	0.108ns
	Soil Amendment Treatments (T)	0.047*
	S x T	0.986ns

***p < 0.001, **p<0.01, *p < 0.05, ns: non-significant at p >0.05.

The individual applications of AMF, BC and CP induced an increase in SDW and RDW in both saline and non-saline soils (**Figure 2C, D**) Additionally, the AMF+BC-treated plants showed a significant increase in their dry matter content for both the shoot and root system of corn plants grown in saline (SDW = 5.5 ± 0.6 g, RDW = 11.5 ± 0.9 g) and non-saline soil (SDW = 7.9 ± 0.4 g, RDW = 16.2 ± 1.7 g).

Under non-saline conditions, the BSD of corn plants amended with AMF+BC showed a 34% increase from the control plants (**Figure 2E**, **Table 3**). However, while the increase in BSD of AMF+BC treated plants, averaging ~4.93 ± .45, showed no significant difference to the control plants under non-saline conditions, it was still notably higher. Likewise, the LP of corn plants under saline and non-saline conditions showed no significant difference with all the amended treatments (**Figure 2F**, **Table 3**). However, the application of bioagents, including AMF, BC, CP, AMF+BC and AMF+CP induced a notable increase in the LP of both saline and non-saline plants. The highest LP, averaging at ~11 ± 1.3 leaves/plant, was observed in the BC-treated plants grown in non-saline soil, followed by ~10 leaves/plant for the AMF+BC and AMF+CP amended plants under both saline and non-saline conditions.

### 3.3 Photosynthetic pigments

**Figure 3:**
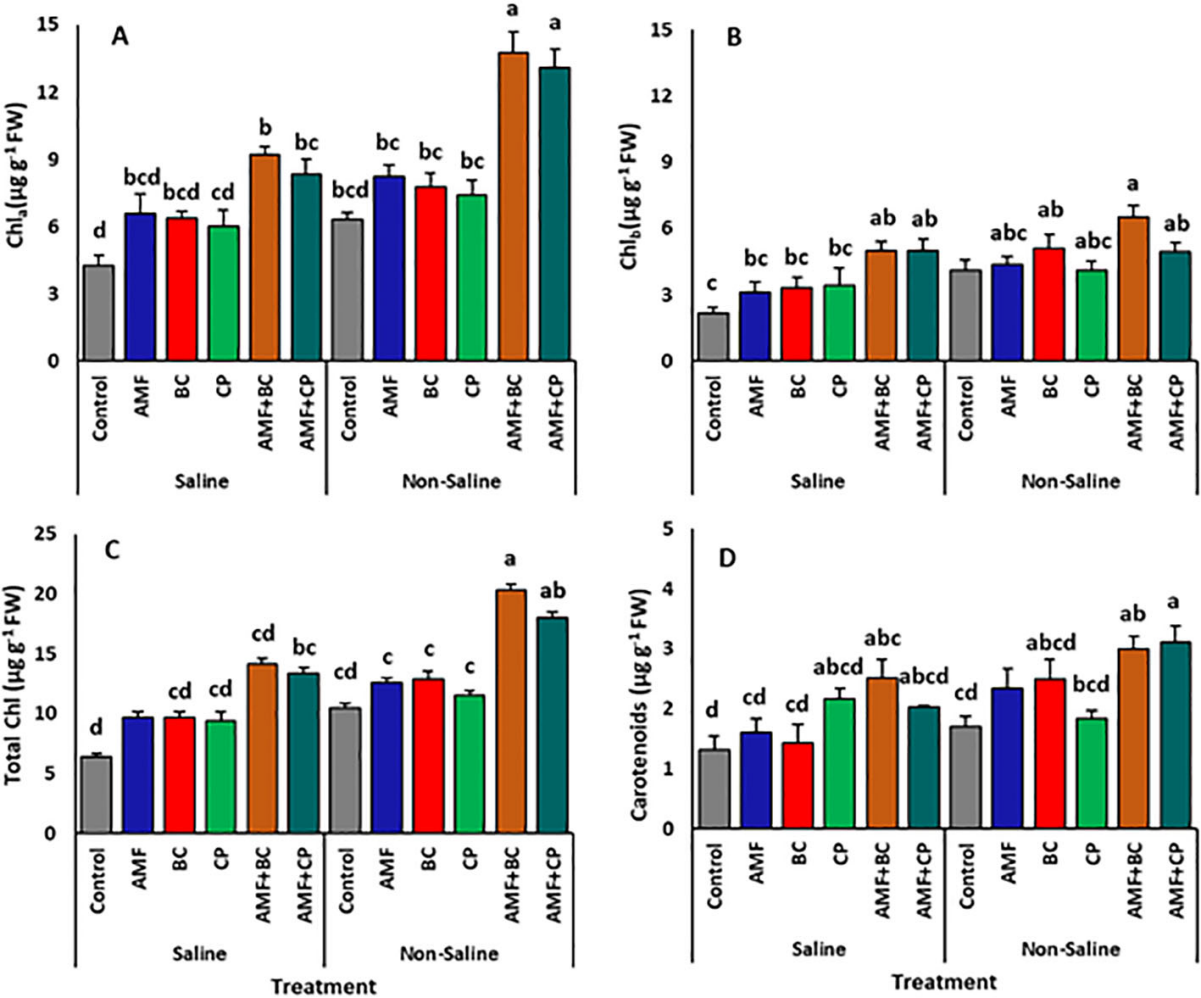
Effects of arbuscular mycorrhizal fungi (AMF), biochar (BC), compost (CP), and their combinations (AMF+BC and AMF+CP) on photosynthetic pigments of maize grown in saline and non-saline soils: **(A)** chlorophyll a (Chl a), **(B)** chlorophyll b (Chl b), **(C)** total chlorophyll (Total Chl), and **(D)** carotenoids. Bars represent mean values ± standard error (n = 4). Different letters indicate significant differences among treatments according to Tukey’s HSD test (p ≤ 0.05).

The photosynthetic pigments chlorophyll*a*, chlorophyll*b*, total chlorophyll and carotenoids of corn plants showed a sharp decline in saline soil compared to the pigments of plants in non-saline soil (**Table 4**). However, the application of AMF, BC, CP, AMF+BC and AMF+CP induced a significant buildup of all these pigments in the plants of both saline as well as non-saline soil (**Figure 3A–D**). The average concentrations of chlorophyll _a_, chlorophyll _b_ and total chlorophyll contents followed similar trends with the application of bioagents (AMF, BC, CP, AMF+BC and AMF+CP). However, the highest production of pigment contents was shown by the plants inoculated with AMF+BC in non-saline (chl_a_ = 13.8 ± 0.9 µg g-1 Fw, chl_b_ = 6.5 ± 0.5 µg g^-1^ Fw, and total chl = 20.3 ± 1.4 µg g^-1^ Fw) soils.

**Table 4:** Analysis of variance (factorial design) of photosynthetic pigments of maize plants growing in saline and non-saline soil with different amendment treatments of AMF, BC, CP, and their combination.

Photosynthetic pigments	Source of variation (ANOVA)	P value
Chla (µg g^-1^FW)	Soil Type (S)	<0.0001***
	Soil Amendment Treatments (T)	<0.0001***
	S x T	0.018*
Chlb(µg g^-1^FW)	Soil Type (S)	0.0002***
	Soil Amendment Treatments (T)	0.0001***
	S x T	0.318ns
Tot Chl (µg g^-1^FW)	Soil Type (S)	<0.0001***
	Soil Amendment Treatments (T)	<0.0001***
	S x T	0.432ns
Carotenoids (µg g^-1^FW)	Soil Type (S)	0.0002***
	Soil Amendment Treatments (T)	0.0001***
	S x T	0.070*

***p < 0.001, *p < 0.05, ns: non-significant at p >0.05.

Under non-saline conditions, the plants treated with AMF+CP showed the highest increase in carotenoids followed by AMF+BC treated plants, averaging ~3.12 ± 0.3 µg g^-1^ FW and ~3.01 ± 0.2 µg g^-1^ Fw, respectively (**Figure 3D**). However, under saline conditions, a significant dip of ~4.8% was recorded in the concentration of carotenoids of AMF+CP treated plants, averaging ~ 2.03 ± 0.03 µg g^-1^ Fw. The highest carotenoid concentration for the plants grown in saline soil was shown at AMF+BC treatment, which averaged ~ 2.52 ± 0.3 µg g^-1^ FW.

### 3.4 Nutrient uptake and sodium accumulation

**Figure 4:**
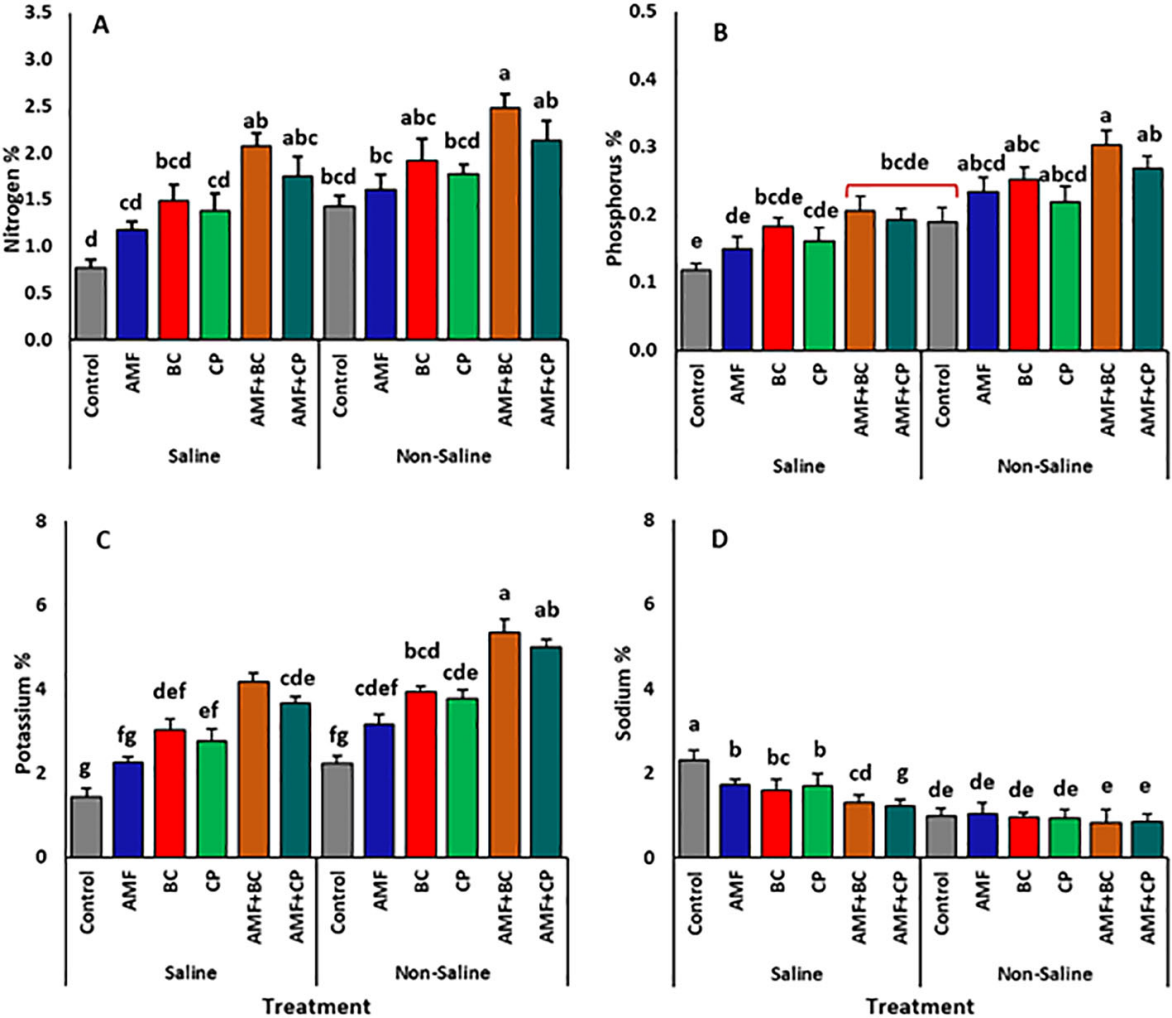
Effects of arbuscular mycorrhizal fungi (AMF), biochar (BC), compost (CP), and their combinations (AMF+BC and AMF+CP) on nutrient content of maize grown in saline and non-saline soils: **(A)** nitrogen (N), **(B)** phosphorus (P), **(C)** potassium (K), and **(D)** sodium (Na). Bars represent mean values ± standard error (n = 4). Different letters indicate significant differences among treatments according to Tukey’s HSD test (p ≤ 0.05).

The corn plants grown in the saline soil exhibited a considerable decline in the NPK levels compared to those harvested from non-saline soils (**Figure 4A–C**; **Table 5**). Yet, nutrient absorption was improved when AMF, BC, CP, and their combinations (AMF+BC and AMF+CP) were added to both saline and non-saline soils. Applying AMF, BC, and CP individually led to a substantial increase in NPK absorption, with BC resulting in notably higher N% (**Figure 4A**), P% (**Figure 4B**) and K% (**Figure 4C**) levels, averaging ~1.49 ± 0.2%, ~0.18 ± 0.01%, and ~3.03 ± 0.2% in saline soils, and ~1.92 ± 0.2%, ~0.25 ± 0.02%, and ~3.94 ± 0.1% in non-saline soils. The maximum boost in the absorption of NPK was exhibited by the plants treated with the combination of AMF+BC followed by AMF+CP in non-saline soils. For example, in non-saline soils, the average N%, P%, and K% of AMF+BC treated plants averaged ~2.48 ± 0.2%, 0.3 ± 0.02%, and 5.34 ± 0.3%, respectively. The similar trend was observed in saline soils.

**Table 5:** Analysis of variance (factorial design) of nutrient elements of maize plants growing in saline and non-saline soil with different amendment treatments of AMF, BC, CP, and their combination.

Nutrients	Source of variation (ANOVA)	P value
Nitrogen%	Soil Type (S)	<0.0001***
	Soil Amendment Treatments (T)	<0.0001***
	S x T	0.957ns
Potassium%	Soil Type (S)	0.026*
	Soil Amendment Treatments (T)	<0.0001***
	S x T	0.569ns
Phosphorous%	Soil Type (S)	<0.0001***
	Soil Amendment Treatments (T)	<0.0001***
	S x T	0.956ns
Sodium %	Soil Type (S)	<0.0001***
	Soil Amendment Treatments (T)	<0.0001***
	S x T	<0.0001***
K: Na Ratio	Soil Type (S)	<0.0001***
	Soil Amendment Treatments (T)	<0.0001***
	S x T	0.132ns

***p < 0.001, *p < 0.05, ns: non-significant at p >0.05.

The Na content of corn plants showed a rapid increase in the saline soils compared to the plants in non-saline soils (**Table 5**). The percentage Na of control plants in non-saline soils accounted for ~0.99 ± 0.05%, which showed a ~32% boost in the saline soils, reaching 2.31% in control saline plants (**Figure 4D**). The introduction of AMF, BC and CP induced a decline in the uptake of Na in both saline and non-saline soils. Notably, the combined application of AMF+CP induced a significant decline in Na absorption, reducing it to ~1.23 ± 0.09% compared to the control plants in saline soils.

**Figure 5:**
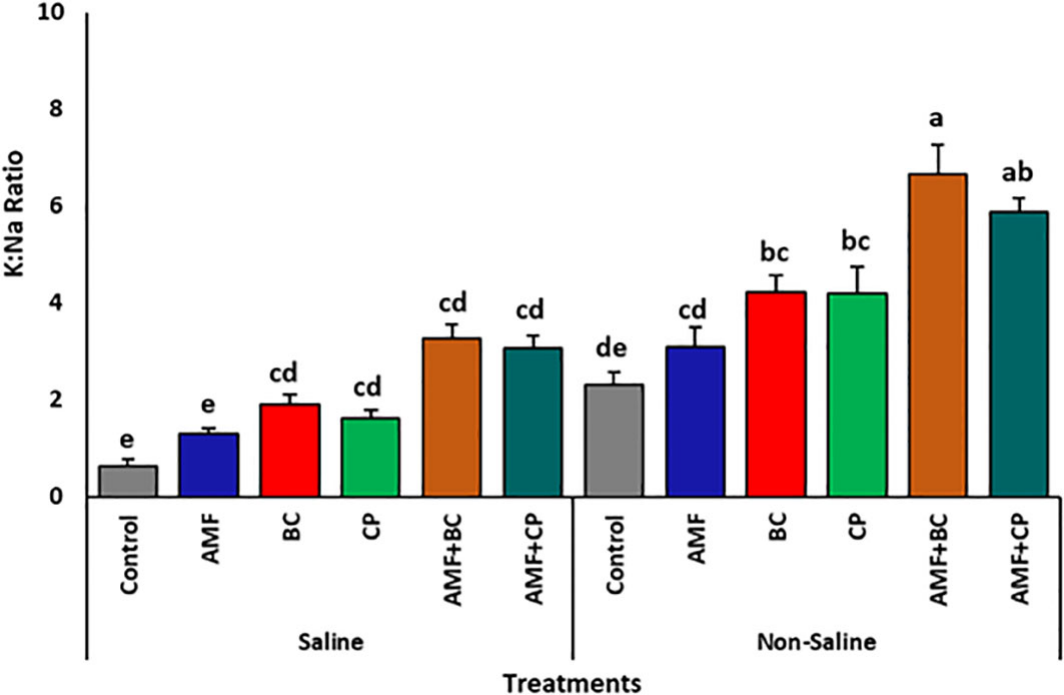
Effects of arbuscular mycorrhizal fungi (AMF), biochar (BC), compost (CP), and their combinations (AMF+BC and AMF+CP) on K:Na ratio of maize grown in saline and non-saline soils. Bars represent mean values ± standard error (n = 4). Different letters indicate significant differences among treatments according to Tukey’s HSD test (p ≤ 0.05).

Soil type and amendments showed significant effect on K:Na ratio (**Table 5**). The K:Na ratio of grown in non-saline soil was higher compared to that in saline soil (**Figure 5**). The application of AMF+BC amendments showed a general increase in K:Na ratio in both non-saline and saline soils compared to their respective controls. For example, K:Na ratio in non-saline soil treated with AMF+BC averaged at ~6.6 ± 0.6%, which is approximately 2.8 times higher than that observed in non-treated control soil, while in saline soils, the K:Na ratio of AMF+BC treatment averaged at ~3.27 ± 0.3%, which is ~5 times higher than its relative control.

### 3.5 Proline content

**Figure 6:**
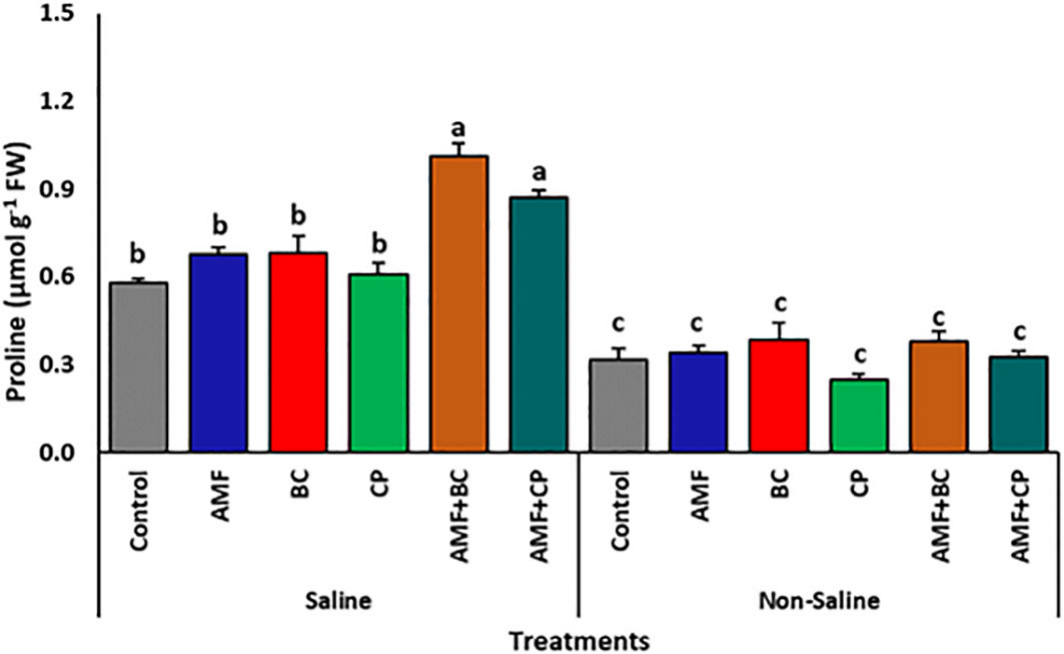
Effects of arbuscular mycorrhizal fungi (AMF), biochar (BC), compost (CP), and their combinations (AMF+BC and AMF+CP) on proline content in maize grown in saline and non-saline soils. Bars represent mean values ± standard error (n = 4). Different letters indicate significant differences among treatments according to Tukey’s HSD test (p ≤ 0.05).

The cultivation of corn in saline soil significantly increased the production of proline as compared to the plants in non-saline soil (**Table 6**). Application of AMF, BC and CP further enhanced the production of proline (**Figure 6**). Notably, the application of AMF combined with BC to the corn plants significantly enhanced the production of proline under saline conditions. For example, AMF+BC treated plants indicated a proline accumulation of 1.01 ± 0.04 µg g^-1^ Fw, which is ~1.8% higher than the control. On the other hand, in non-saline soils, the application of AMF, BC and CP individually or AMF combined with BC or CP showed no significant difference on the proline content of plants.

**Table 6:** Analysis of variance (factorial design) of proline content, oxidative damage and antioxidant activity of maize plants growing in saline and non-saline soil with different amendment treatments of AMF, BC, CP, and their combination.

Biochemical parameters	Source of variation (ANOVA)	*P value*
Proline (µmol g^-1^ FW)	Soil (S)	<0.0001***
	Soil Amendment Treatments (T)	<0.0001***
	S x T	0.0001***
SOD (Umg^-1^ FW)	Soil (S)	0.001**
	Soil Amendment Treatments (T)	0.830ns
	S x T	0.907ns
CAT (U mg^-1^ FW)	Soil (S)	<0.0001***
	Soil Amendment Treatments (T)	0.011*
	S x T	0.070*
APX (U mg^-1^ FW)	Soil (S)	<0.0001***
	Soil Amendment Treatments (T)	0.739ns
	S x T	0.974ns
POD (U mg^-1^ FW)	Soil (S)	<0.0001***
	Soil Amendment Treatments (T)	0.030*
	S x T	0.317ns
MDA(µmol g^-1^ FW)	Soil (S)	<0.0001***
	Soil Amendment Treatments (T)	<0.0001***
	S x T	<0.0001***
H_2_O_2_(µmol g^-1^ FW)	Soil (S)	<0.0001***
	Soil Amendment Treatments (T)	<0.0001***
	S x T	<0.0001***

***p < 0.001, **p<0.01, *p < 0.05, ns: non-significant at p >0.05.

### 3.6 Biochemical traits and antioxidant enzyme activity

**Figure 7:**
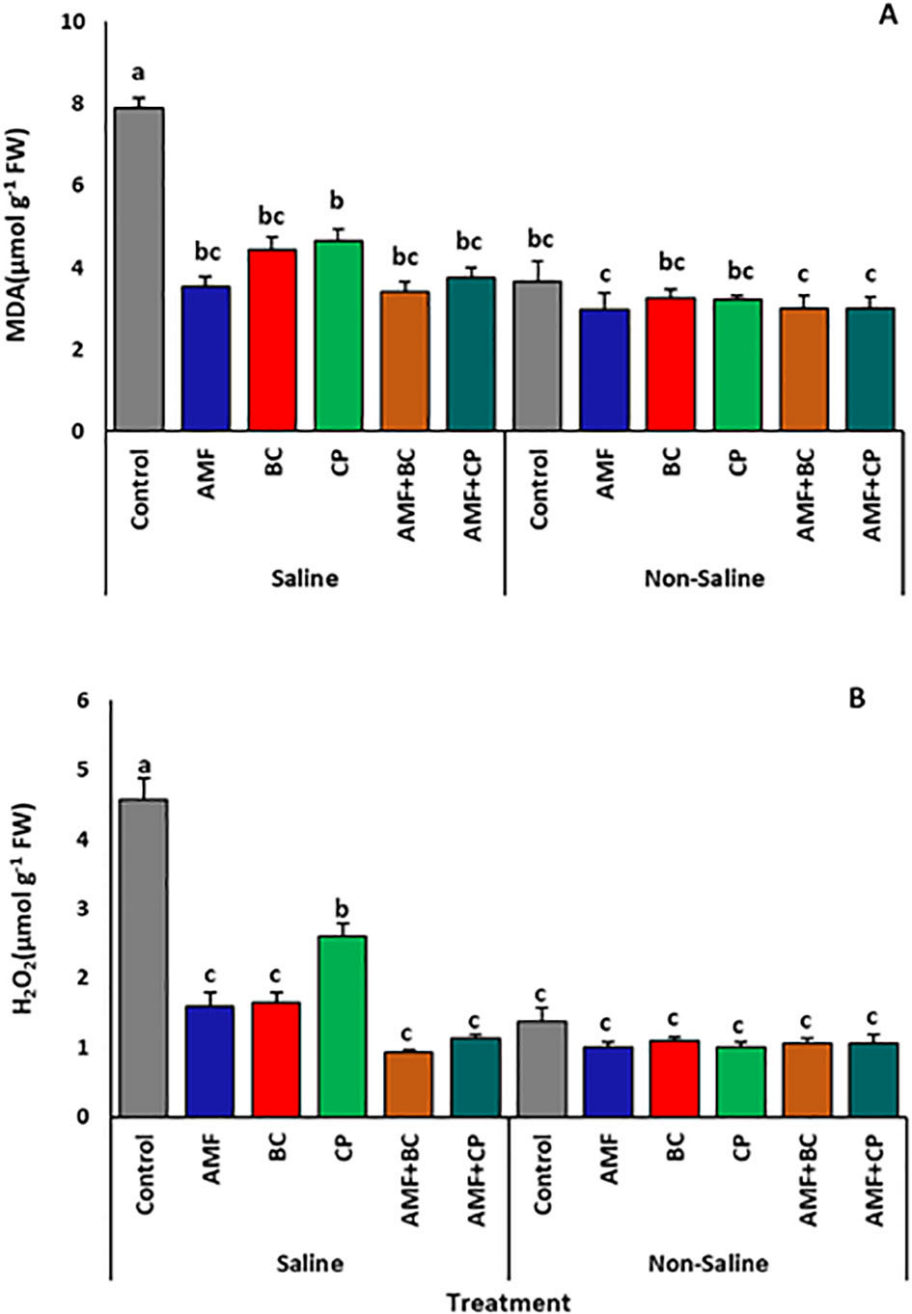
Effects of arbuscular mycorrhizal fungi (AMF), biochar (BC), compost (CP), and their combinations (AMF+BC and AMF+CP) on oxidative stress indicators in maize grown in saline and non-saline soils: **(A)** malondialdehyde (MDA) and **(B)** hydrogen peroxide (H₂O₂). Bars represent mean values ± standard error (n = 4). Different letters indicate significant differences among treatments according to Tukey’s HSD test (p ≤ 0.05).

Statistical analysis of data indicated a significant impact of soil salinization on the MDA contents of corn plants (**Table 6**). Control plants grown in saline soils showed a significant accumulation of MDA content, averaging ~7.88 ± 0.2 µmol g^-1^FW, 16.9% higher than the control plants under non-saline conditions which averaged ~3.66 ± 0.2 µmol g^-1^FW (**Figure 7A**). The amendment of AMF, BC, CP and AMF combined with BC or CP significantly reduced the production of MDA. In saline soils, the AMF+BC treated plants showed the maximum decrease in MDA concentration (averaging 3.41 ± 0.2 µmol g^1^ FW), approximately half that of the control.

The H_2_O_2_ activity followed a similar trend as that of MDA in saline as well as in non-saline soils (**Table 6**). The H_2_O_2_ content of corn plants in saline soils was significantly higher than the plants grown in non-saline soil, with non-inoculated control plants of saline soil showing H_2_O_2_ levels about five times higher than non-inoculated control plants grown in non-saline soil (**Table 3**). The application of AMF, BC, CP and AMF combined with BC or CP proved effective in reducing H_2_O_2_ accumulation in the corn plants grown in saline soil (**Figure 7B**). The highest decline in H_2_O_2_ activity was observed in the AMF+BC treated plants averaging 0.92 ± 0.03 µmol g^1^ FW.

**Figure 8:**
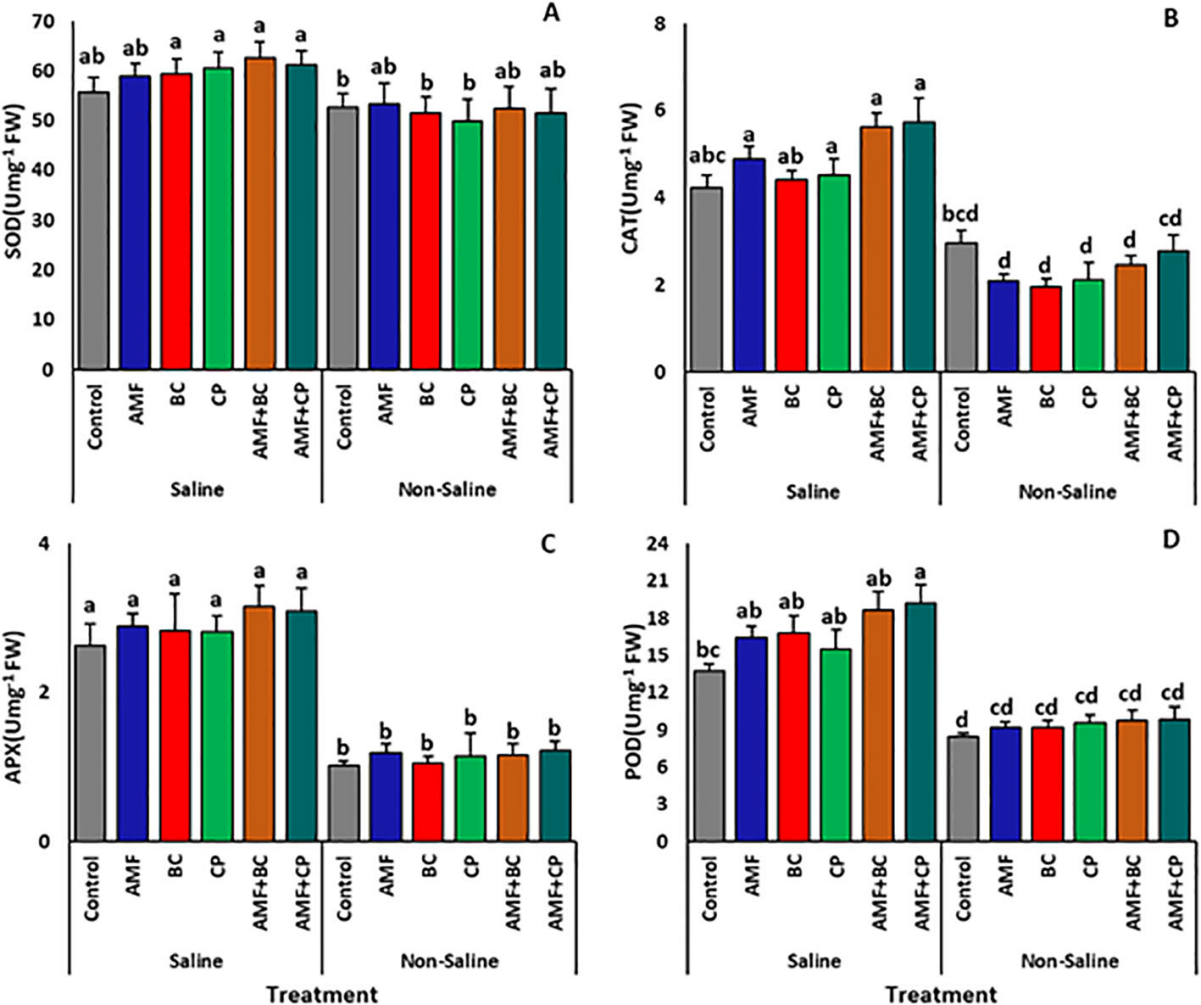
Effects of arbuscular mycorrhizal fungi (AMF), biochar (BC), compost (CP), and their combinations (AMF+BC and AMF+CP) on antioxidant enzyme activities in maize grown in saline and non-saline soils: **(A)** superoxide dismutase (SOD, EC 1.15.1.1), **(B)** catalase (CAT, EC 1.11.1.6), **(C)** ascorbate peroxidase (APX, EC 1.11.1.11), and **(D)** peroxidase (POD, EC 1.11.1.7). Bars represent mean values ± standard error (n = 4). Different letters indicate significant differences among treatments according to Tukey’s HSD test (p ≤ 0.05).

The antioxidant enzyme activities including SOD, CAT, APX and POD are shown in **Figure 8A–D**. The maize grown in saline soils significantly increased the SOD, CAT, APX and POD activities compared to the that in non-saline soils (**Table 3**). The inoculation of the plants in saline conditions further enhanced the enzyme activity with no significant difference among treatments. Nevertheless, the application of AMF+BC induced a maximum SOD activity (62.66 ± 3.3 U mg^-1^ FW) in corn plants against the oxidative damage of salinity stress followed by AMF+CP-treated plants (61.3 ± 2.7 U mg^-1^ FW) (**Figure 8A**). The inoculation of plants with AMF showed an improved activity of CAT compared to the control plants under saline stress, while the effect of BC and CP treatments was slightly lower (**Figure 8B**). Of all the treatments, the combined application of AMF+CP induced the maximum mitigating effects on the plants under salinity stress by significantly improving the CAT activity which averaged 5.74 ± 2.7 U mg^-1^ FW, followed by AMF+BC with CAT activity of 5.63 ± 2.7 U mg^-1^ FW. In non-saline soil, the highest CAT activity (2.96 ± 0.3 U mg^-1^ protein) was recorded in non-inoculated control plants which showed a decline with the application of stress-mitigating treatments. Further, the mitigating effects of AMF, BC, CP and AMF combined with BC or CP on APX content showed no significant difference with one another in either saline or non-saline soils (**Figure 8C**). However, under salinity stress, all the treatments induced a slightly insignificant increase in the APX activity compared to the control plants. In the case of POD, the soil salinization induced a significant increase in the POD activities which was further enhanced by the introduction of AMF, BC, CP, AMF+BC and AMF+CP (**Figure 8D**, **Table 6**). However, the POD activities in non-saline soil showed no significant difference among the applied treatments. In the saline soil, the POD activity of control plants was 22% higherthan the control corn plants in non-saline soil. Amendments of saline soil with AMF, BC, CP and AMF combined with BC or CP further boosted the POD content. The maximum POD activity was recorded in AMF+CP which was 27% higher, followed by a 25% increase in AMF+BC-treated plants than the control.

**Figure 9:**
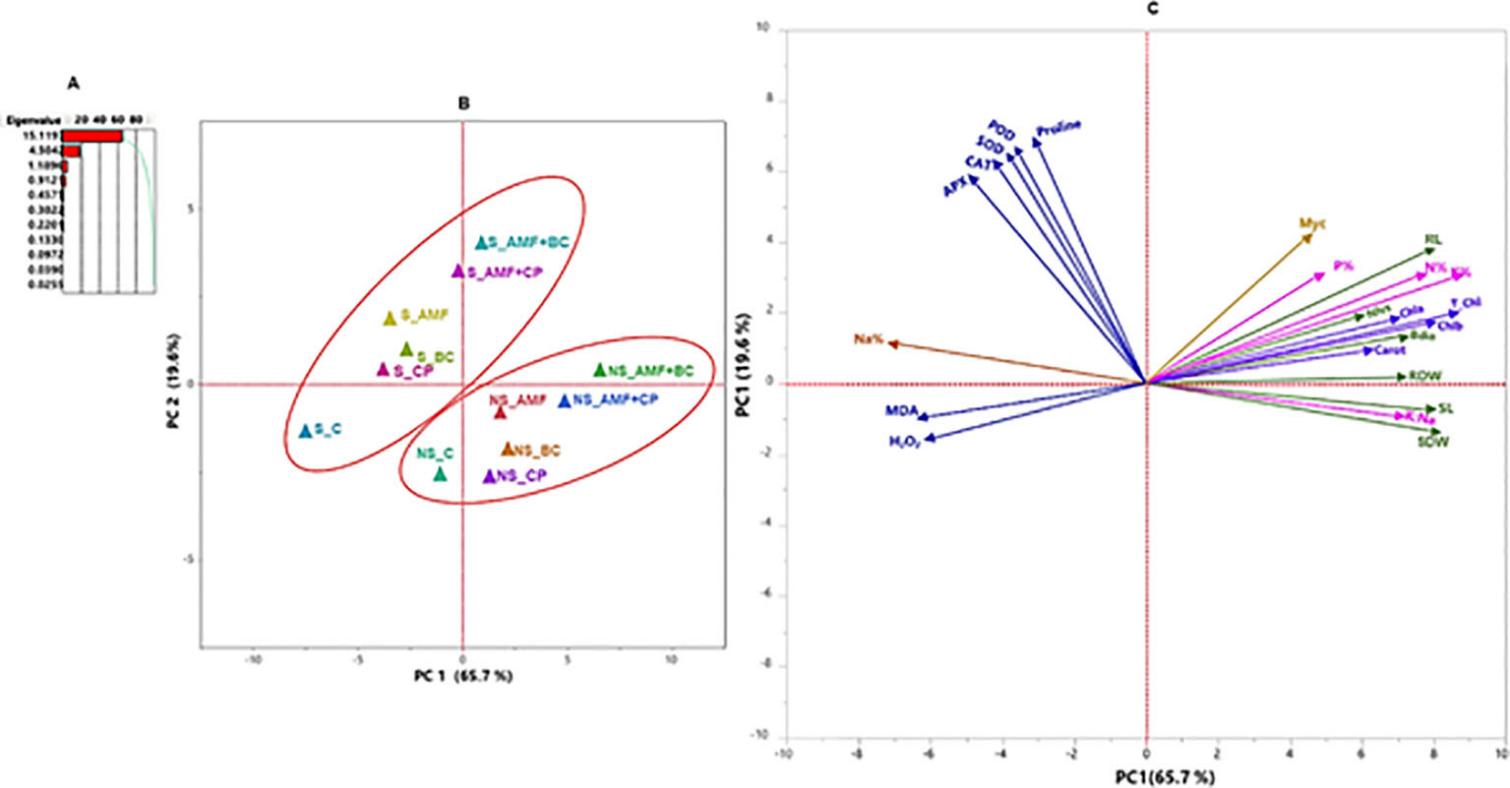
Principal component analysis (PCA) of growth, physiological, nutrient, and biochemical parameters of maize grown in saline and non-saline soils: **(A)** eigenvalues, **(B)** treatment distribution, and **(C)** trait distribution. Treatment abbreviations: S_C (saline control), S_AMF, S_BC, S_CP, S_AMF+BC, S_AMF+CP, NS_C (non-saline control), NS_AMF, NS_BC, NS_CP, NS_AMF+BC, and NS_AMF+CP. Trait abbreviations: SL, shoot length; RL, root length; SDW, shoot dry weight; RDW, root dry weight; L/P, leaves per plant; Chl a, chlorophyll a; Chl b, chlorophyll b; Total Chl, total chlorophyll; carot, carotenoids; N, nitrogen; P, phosphorus; K, potassium; Na, sodium.

Principal component analysis (PCA) incorporated all the measured traits of all the bio-inoculant treatments. Two main components that accounted for 87.7% of total variability (PC1 = 68.7% and PC2 = 19% were observed (**Figure 9**). Under non-saline conditions, PCA revealed a highly interrelated positive correlation among bio-inoculant treatments (applied singly or combined) with growth parameters, nutrient content (N, P, K, and K:Na ratios), and photosynthetic pigments. The analysis also confirmed the adverse effect of saline soil on these parameters. The biplot positive correlation between saline soil and higher Na^+^, MDA, proline and H_2_O_2_ levels. PCA showed a strong positive correlation between the combined application of AMF with BC and CP and the antioxidant system (antioxidant enzymes and osmolytes) in saline soil.

## 4 Discussion

Soil salinity is a key constraint to maize productivity, primarily due to its disruptive effects on plant growth, nutrient uptake, and physiological functioning. In the present study, salinity significantly reduced maize biomass and photosynthetic pigment content while increasing sodium accumulation and oxidative stress indicators. These responses are well documented in maize and other glycophytic crops, where salinity induces ionic imbalance, osmotic stress, and excessive generation of reactive oxygen species (ROS), ultimately impairing plant performance (Munns and Tester, 2008; Farooq et al., 2015; Islam et al., 2023).

Application of arbuscular mycorrhizal fungi (AMF), biochar (BC), and compost (CP), either individually or in combination, alleviated several salinity-induced constraints. Improvements in shoot and root growth observed in these treatments can be attributed to enhanced nutrient availability, improved soil physicochemical conditions, and AMF-mediated expansion of the effective root absorption zone. Similar growth promoting effects of AMF under saline conditions have been reported in maize and other crops, largely due to improved acquisition of phosphorus and potassium and better water relations (Parihar et al., 2020; Malik et al., 2024; Peng et al., 2024).

Among the tested treatments, the combined application of AMF with biochar consistently produced superior responses compared with individual amendments or AMF combined with compost. Biochar’s high porosity, surface area, and ion-sorption capacity enhance nutrient retention while reducing sodium bioavailability in saline soils (Blackwell et al., 2010; Mbarki et al., 2018; Nguyen et al., 2022). When combined with AMF symbiosis, these properties promote improved nutrient uptake and ion homeostasis, as reflected by the higher K:Na ratio observed in this study. Maintenance of a favorable K:Na ratio is a key determinant of salt tolerance, as potassium plays a central role in enzyme activation, osmotic adjustment, and membrane stability, whereas excess sodium disrupts these processes (Shabala and Pottosin, 2014; Chakraborty et al., 2016).

Salinity stress markedly increased proline accumulation and oxidative damage indicators, including malondialdehyde (MDA) and hydrogen peroxide (H_2_O_2_). The reduction of these stress markers in AMF and amendments treatments suggests improved cellular redox regulation. Enhanced activities of antioxidant enzymes in combined treatments indicate a coordinated stress-mitigation response that limits lipid peroxidation and cellular injury. Similar enhancements of antioxidant enzyme activity under AMF and organic amendments application have been reported in maize, wheat, tomato, and date palm under saline conditions (Latef and Chaoxing, 2011; Akhtar et al., 2015; Ait-El-Mokhtar et al., 2019; Han et al., 2024).

The principal component analysis further supported these findings by demonstrating strong positive associations between combined amendment treatments and growth parameters, nutrient balance, and antioxidant activity, whereas salinity stress was closely associated with sodium accumulation and oxidative damage. Collectively, these results indicate that synergistic interactions between AMF and organic amendments, particularly biochar, enhance maize tolerance to salinity through integrated effects on nutrient acquisition, ion regulation, and oxidative stress mitigation, consistent with earlier reports on AMF–biochar interactions under abiotic stress (Ait-El-Mokhtar et al., 2020; Gunes et al., 2023; Ouhaddou et al., 2022).

## 5 Conclusion

Soil salinity adversely impacted the growth of corn through alteration in the morphological, physiological and biochemical parameters. Higher concentrations of Na ions lead to ROS-mediated oxidative damage, which results in the decline of photosynthetic pigments. This study demonstrates that arbuscular mycorrhizal fungi (AMF), biochar (BC), and compost (CP) can alleviate salinity-induced stress, with the combined application of AMF and organic amendments, particularly biochar providing the most consistent improvements. The AMF+BC treatment enhanced biomass production, increased uptake of essential nutrients, reduced sodium accumulation, improved the K:Na ratio, and strengthened antioxidant defense mechanisms, thereby limiting oxidative damage under saline conditions.

Therefore, a combination of AMF with BC or CP has a potential application as an eco-friendly and cost-effective tool in alleviating the impact of salinity stress on plants grown in salt-affected soil. While the results were generated under controlled greenhouse conditions, they provide a clear framework for developing integrated strategies for salinity management, warranting further validation under field conditions.

## Data Availability

The raw data supporting the conclusions of this article will be made available by the authors, without undue reservation.
