# Supplementary material for: Interactive effects of arbuscular mycorrhizal fungi and organic amendments on maize growth under salinity stress
Source: Front Plant Sci. 2026 Mar 6;17:1638742. doi: 10.3389/fpls.2026.1638742 (PMC13002420; doi:10.3389/fpls.2026.1638742)
Supplement: Supplementary file 1 [file SupplementaryFile1.docx]

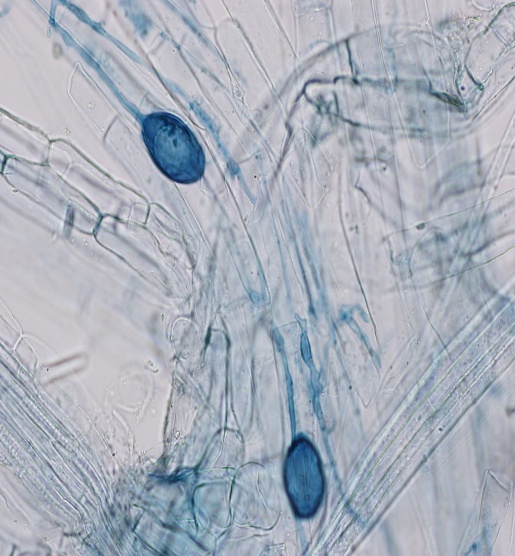

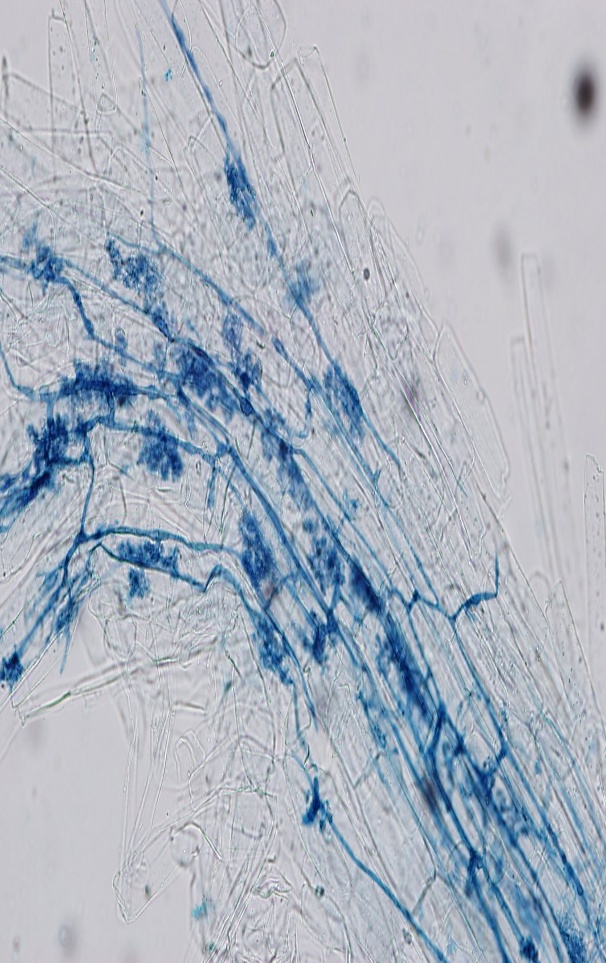

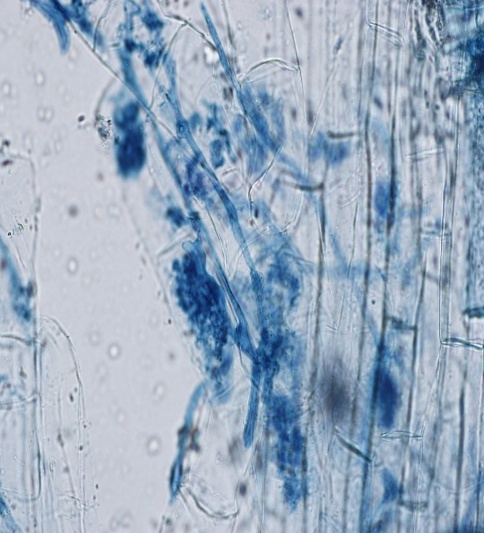

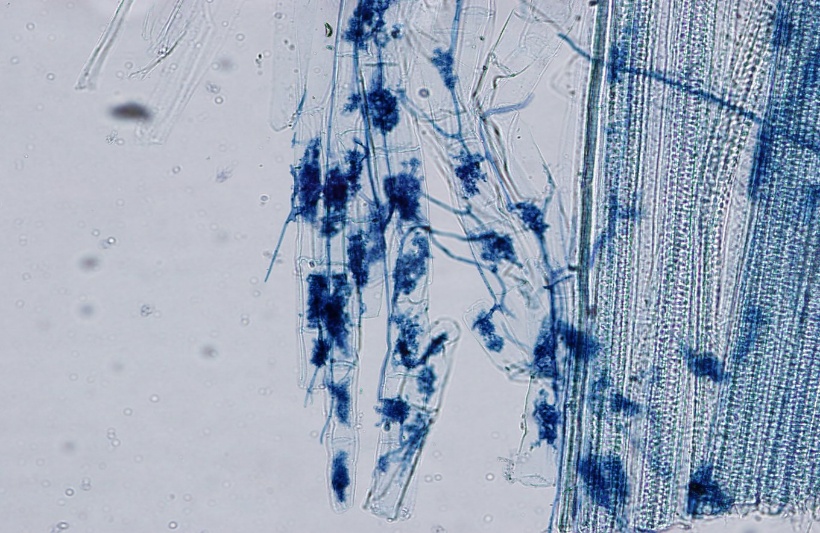

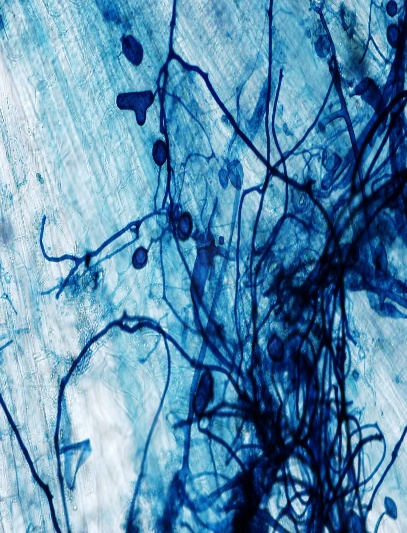

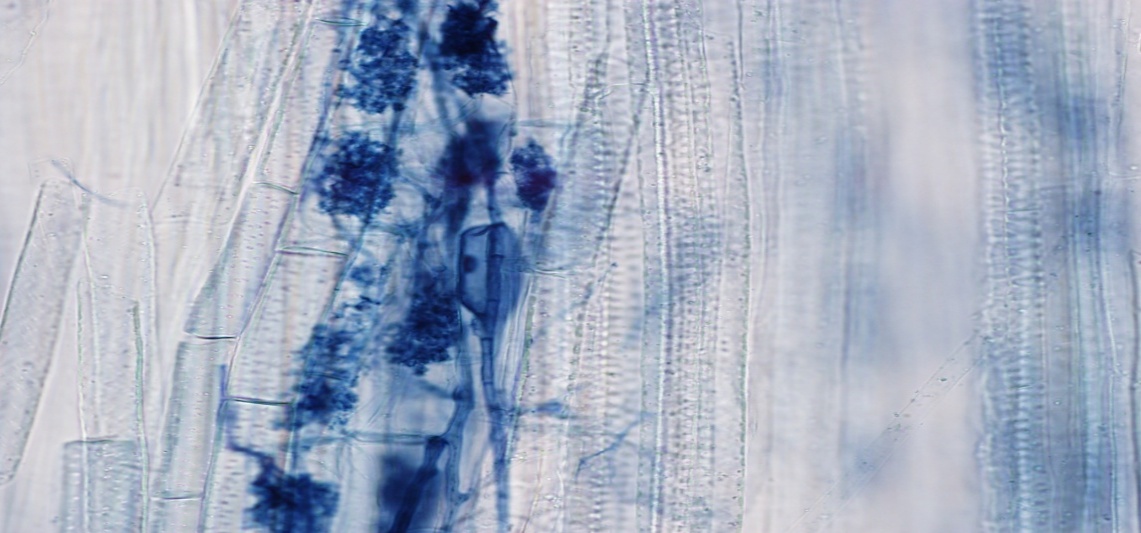


**a**

**b**

**c**

**f**

**e**

**d**

**Saline Soil**

**Non-Saline Soil**

Supplementary Figure: Microphotographs showing the colonization in the roots of maize in saline (a-c) and non-saline soils (d-f). (a & d) AMF treated roots, (b & e) AMF+BC treated roots and (c & f) AMF+CP treated roots.
